# Measuring the Psychological Distance between an Organization and Its Members—The Construction and Validation of a New Scale

**DOI:** 10.3389/fpsyg.2017.02296

**Published:** 2018-01-09

**Authors:** Hong Chen, Shanshan Li

**Affiliations:** School of Management, China University of Mining and Technology, Xuzhou, China

**Keywords:** employee, organization, psychological distance, scale development, qualitative analysis, quantitative analysis

## Abstract

There exists a lack of specific research methods to estimate the relationship between an organization and its employees, which has long challenged research in the field of organizational management. Therefore, this article introduces psychological distance concept into the research of organizational behavior, which can define the concept of psychological distance between employees and an organization and describe a level of perceived correspondence or interaction between subjects and objects. We developed an employee-organization psychological distance (EOPD) scale through both qualitative and quantitative analysis methods. As indicated by the research results based on grounded theory (10 employee in-depth interview records and 277 opening questionnaires) and formal investigation (544 questionnaires), this scale consists of six dimensions: experiential distance, behavioral distance, emotional distance, cognitive distance, spatial-temporal distance, and objective social distance based on 44 items. Finally, we determined that the EOPD scale exhibited acceptable reliability and validity using confirmatory factor analysis. This research may establish a foundation for future research on the measurement of psychological relationships between employees and organizations.

## Introduction

Along with the development of the global economy, marketing competition has intensified and the value of human resource has gradually been highlighted, which have brought complex employment relationship challenges to many companies. A survey on China’s employment relationships demonstrated that over 50% of employees thought that their bosses were unreliable; 56% of managers and 64% of staff members thought of quitting approximately 12 times per year; 38% of managers and 47% of staff members were not satisfied with their present work (Allinson et al., 2001). An increasing number of managers are becoming confused with the existing employee-organization relationships. However, healthy employee-organization relationships are strategically important to the healthy development of an organization. Employee-organization relationship is a type of social exchange relationship between an organization’s inducements on employers and employees’ contributions to the organization, including economical, social and psychological factors (Tsui et al., 1995; Chen et al., 2005). From the perspective of employer, the relationship between organization and employee is taken as the social exchange relationship between organization’s input for employee and employee’s return for organization; From the perspective of employee, scholars describe the relationship between employee and organization based on organization commitment and express “the total intensity of accepting and being involved into a specific organization of an individual” (Porter et al., 1974). Although numerous studies concerning employee-organization relationship have been conducted, there still exist certain limitations.

First of all, employee-organization internal driving force and external driving force results present the “either-or” separation in the long run. At present, most studies concerning organization commitment focus on attitudes and emotions and refer to the emotional attachment established by employee and organization based on sharing values and interests. It emphasizes that effective organization commitment relies more on work features rather than personal factors and proposes to take organization commitment as a concept of external driving force rather than internal driving force (Hallberg and Schaufeli, 2006). Whereas, the advantage or disadvantage of employee-organization relationship is the combined function of external work features and personal factors. Therefore, this study derives the conclusion that organization commitment could not precisely describe employee-organization relationship. Secondly, existing employee-organization relationship studies mostly center around indirect variables such as work engagement (Deligero and Laguador, 2017), identification with the organization (Lu and Torng, 2017), organization of citizen behaviors (Chin, 2015; Koning and Kleef, 2015). For instance, Rich et al. (2010) demonstrate that work engagement is the concept which connects individual personality with organization factor and work performance. Consequently, work engagement is usually regarded as a regulated variable in the study on the relationship between employee and organization (Chang et al., 2013; Kataria et al., 2013). Although these indirect organization variables could reflect the behaviors of employees in organization context, they fail to precisely and explicitly reveal the distance of the practical relationship between employee and organization. Prediction of employee-organization relationship has always been the difficulty in the research field of organization management. Thirdly, existing employee-organization relationship studies all use employees’ unitary emotional sense of belonging toward organization to judge the intimacy between employee and organization. For instance, identification with the organization is individual cognitive process toward organization sense of belonging which manifests the consistency between individual and organization in the aspect of values (Ashforth and Mael, 1989; Knippenberg and Hogg, 2003). Identification with the organization has the characteristics of persistent emotional bond (Gioia et al., 2000). Whereas, since all human emotions are inseparable from realistic relationship, the integration of different psychological relationship and realistic relationship has become the tendency of employee-organization relationship study.

Accordingly, this study intends to raise variables that could directly perceive the relationship between employee and organization based on internal driving force and external driving force concept and integration of psychological relationship and realistic relationship.

In natural science, distance refers to the length of time or space between specific objects. In 1912, Edward Bullough, a Swiss psychologist, developed the concept of psychological distance in the esthetics field to illustrate that esthetic feelings stemmed from the psychological distance that an observer perceived between himself/herself and artwork. In recent years, psychological distance has been viewed as a kind of pure perspective orientation (Dhar and Kim, 2007; Sun et al., 2007). This concept emphasized the importance of an individual’s perception and understanding of his/her environment, its central point being that individuals’ reactions to events depend on their mental representation of the matter (Liberman et al., 2002; Nussbaum et al., 2003; Bar-Anan et al., 2007). Psychological distance is defined in psychological terms as “the degree of emotional bonding between people in the process of interpersonal communication” (Wu and Bai, 2015). Based on the two understandings of psychological distance, we can see that due to the difference of individual personality and personal construct processes, the employee-organization relationship must be self-oriented to first generate the perception and understanding of closeness, then judge and decide on organizational behavior. The judgment will usually manifest as willingness to stay in the organization and dedicate oneself to it. Accordingly, we introduce psychological distance into the field of organization behavioral science and propose the concept of employee-organization psychological distance (EOPD), which can be used to describe the level of perceived correspondence or interaction between subjects and objects. In other words, psychological distance can be viewed as a direct manifestation of the employee-organization relationship, which also provides a possibility to evaluate the relationship. From a practical perspective, extreme incidents, informal turnover and occupational burnout demonstrate psychological alienation between staff and companies and can result in significant losses to organizations. Therefore, EOPD is significant, and it is vital to develop a method to measure this relationship.

Based on the statements above, we introduced psychological distance into the area of organizational management and focused on the development and examination of an EOPD scale. This research included five steps: (1) Based on data obtained through a literature review, interviews and open-ended questionnaires, we used qualitative analysis tools to determine and extract the items for the EOPD scale and construct the original scale. (2) Pre-survey data was used to refine the scale, examine its structure and revise the questionnaire. (3) Formal survey data was analyzed using SPSS to explore the structure of the scale. (4) AMOS was utilized to examine the scale. (5) The reliability and validity of the scale were examined.

Managers can immediately use the EOPD scale to measure employees’ affective status at work and mental closeness to their organization to enhance their management efficiency with less effort. Our research is intended to provide a new perspective on the employee-organization relationship.

## The Current Research

At present most studies of psychological distance are related to construal level theory, which is originated form temporal construal theory (Liberman and Trope, 1998), It is believed that time is a psychological distance, which affects the level of individual interpretation and influences the other cognition and behavior of the individual; Then Liberman et al. (2007) extended the psychological distance from the time distance to the space distance, the social distance and the possibility of occurrence, and finally developed it as the temporal level theory; Trope et al. (2007) proposed the term “psychological distance” to define a construal level and thus enable people to have different cognitive judgment and the “distance” factors for decision making, and based on this, providing the detailed explanation of the relationship between the construal level and psychological distance. Trope and Liberman (2010) formally put forward a unified the psychological distance of construal level theory, which explain people’s reactions to cognitive object cognition and evaluation decision mechanism through introducing the core concept of “psychological distance.”

Construal level theory is a kind of social cognitive theory, which emphasizes the importance of personal perception and understanding of environment, one of its core idea is people’s reactions to social events depends on the mental representation of events (Liberman et al., 2002; Nussbaum et al., 2003), although this perceptual process provides reference for the employee-organizational psychological distance, it does not construct the full picture of interpersonal psychological distance.

The psychological distance is widely used in various research fields, in the field of trade, it mainly refers to hinder or interfere with the flow of information between the enterprise and the target country, and causes some uncertainty factors of enterprise to overseas market, these factors include the differences between enterprise and target countries in culture, language, political system, people’s education level and the degree of industrialization (Håkanson, 2014); In the consumer domain, it aims to explain consumer preferences and consumer behavior choices. In the field of human relations in society, psychological distance is defined as a sense of uncertainty of people in different status, values, and cultural background to the surrounding relationship produced, leading to its intimate or alienated subjective feeling (Wang et al., 2013). Psychological distance research has gradually attracted the attention of scholars and continues to expand into other fields. This research focuses on the psychological distance in the organizational field based on interpersonal perspectives.

Based on the above research on psychological distance, the response of employees to the organization is dependent on the psychological representation of the organization, which is also the core of the employees’ psychological distance. The psychological representation is the result of the common effect of work features (external driving force) and personal factors (internal driving force), and all kinds of emotions do have associations with realistic relationships. In a study of interpersonal relationships, Huang (2015) stated that interpersonal psychological distance was based on actual interpersonal communications, and good interpersonal relationships unify realistic relatedness and psychological relations. Hence, this study aims to combine psychological relations (internal driving force) with realistic relatedness (external driving force) to observe employee-organization relationship.

When observing realistic relationships, physical distance can directly demonstrate the degree of closeness between people. Edward Hall observed the bodily distance kept between oneself and others in communications, and in his research divided interpersonal distance into intimate distance (0–45 cm), personal distance (45–120 cm), common distance (120–360 cm) and public distance (great than 360 cm). This researcher also argued that physical distance developed relative to emotional distance because the compatible relationships differed between various individuals. This principle is similar in organizations, which are systems containing numerous relationships. Although bodily contact does not exist between employees and organizations in the same way, realistic relationships do exist between employees and organizations. The realistic relationship includes factors, such as time, space and society. For instance, time, which in this case refers to the length of the employee’s tenure in the organization, will result in a sense of closeness or distance between employee and organization. In other words, job seniority impacts individuals’ emotional commitment (Allen and Meyer, 1990; Liu, 2011). Space can also cause a sense of closeness or distance. Unlike bodily contact, this kind of employee-organization spatial feeling appears as a sense of geographical belonging. Studies have demonstrated that people experience a sense of belongingness in their hometown (Shi, 2006) and that geographical belongingness can influence people’s employment as well as demission (Ying X.H., 2011). Furthermore, employees possess social attributes, which means that they will develop relationships with colleagues, superiors and subordinates. People with different backgrounds may experience difficulty developing interpersonal attraction to one another (Berscheid and Walster, 1978), while people from similar backgrounds may more easily develop a sense of empathy for one another due to interpersonal similarities. Empathy helps people to understand and predict others’ emotions and behaviors effectively and thus promotes altruistic and cooperative actions (Morishima et al., 2012; Balconi and Canavesio, 2013). Social factors also impact the employee-organization relationship, which therefore demonstrates that realistic relationships are actual reflections of employee-organization relationships. From the perspective of psychological relations, employees have emotional attachment on organization and establish emotional reliance on organization based on sharing values and interests. Besides, employees have also formed some expectations of organizations from judging specific matters and past experiences. These experiences include salary (Li et al., 2016) and promotion opportunities (Kraimer et al., 2011). These expectations can therefore immediately affect employees’ working state and current organization-related relationships.

To conclude, EOPD consists of four parts: spatial distance (regarding the distance between the organization and its members in the dimension of space), temporal distance (regarding the length of the period that each individual spends with the company, either from the present to sometime in the organization’s future or from the organization’s past to the present), social distance (regarding the distance between employees and social factors in organizations), expectation (regarding judgment of future trends or event occurrence using personal experiences), and emotional belonging (regarding the emotional reliance established by employees on organization based on sharing values and interests). Namely, realistic and psychological relationships jointly constitute EOPD.

**FIGURE 1:**
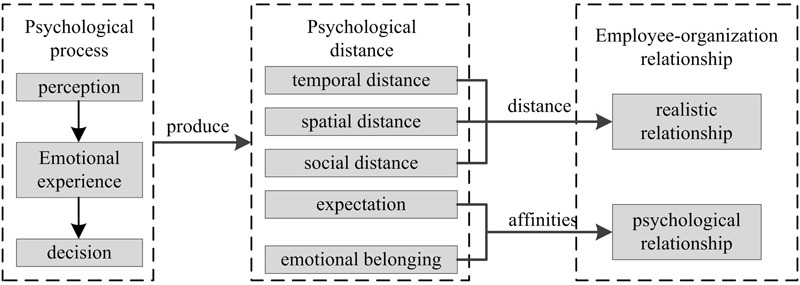
Analytical graph of employee-organization psychological distance.

Viewing the development process of the employee-organization relationship, It is easy to notice the logical relations of individual‘s psychological changes. In the first step, an individual will perceive the distance of the specific object in his/her abstract organizational psychological space (Liberman et al., 2007). Notably, perceptions in this stage are based on consciousness of the object instead of the object itself. This consciousness is the result of information interpretation and processing, which depend not only on the object’s physical attributes but also on the relationship between subject and object. In the second step, based on the consciousness of the first stage, the individual will develop subjective (implicit or explicit) judgments on the self-other (“other” here refers to different people, although they are all others) relationship or self-organization relationship and generate related emotional experiences, which usually manifest as psychological attractions or exclusions (Agnew et al., 2004). In the last step, psychological attraction or exclusion will influence the individual’s decisions regarding behaviors (Trope and Liberman, 2010). These decisions immediately produce the sense of closeness or distance in the aspects of time, space, social expectations and emotional belonging between individuals and their organization, and these senses constitute the realistic and psychological attributes of the employee-organization relationship. Realistic relations and psychological relations are integrated expressions of EOPD, which is a direct manifestation of the employee-organization relationship. The development process is illustrated in **Figure 1**.

Employee-organization psychological distance is our major way of measuring the employee-organization relationship. This distance can be described as an employee’s subjective perception regarding the distance between himself/herself and the organization and is based on the individual level of acceptance and willingness to predict, assess and implement the organization’s mission. EOPD can be used to describe the level of perceived correspondence or interaction between subjects and objects. The straight measurement of employees’ psychological distance can reflect the employee-organization relationship in an intuitive, accurate, all-sided and timely way to gain a higher management efficiency.

## The Measurement Method

Employee-organization psychological distance is a brand new concept, so scales of it did not previously exist. At present, prime measurement methods concerning psychological distance in sociology field include MAPS test (Make a Picture Story), CID test (Comfortable Interpersonal Distance), PDS scale (Psychological Distance Scale), and IOS questionnaire (Relation Closeness Inventory), and of which, MAPS tests estimate inner personal psychological distance by analyzing the spatial distance of the selections and settings of human models (Schaefer and Higgins, 1976). As a projection test, MAPS can test projects relevant information about psychological distance while choosing figure context and recounting the story, whereas, MAPS measurement simply derives space distance. Apart from its complicated operation, this test method has numerous variables. The CID test depends on the imagination of its subjects to describe the spatial distance and to demonstrate the psychological distance (Gottheil et al., 1968). In essence, it measures certain space distance. But its effectiveness remains to be testified. The PDS scale uses a series seven figures in pairs (sister, stranger, father, brother, neighbor, friend, intimate friend, and mother) representing the self and others and transforms their labels into scores that can be used to measure the psychological distance between the subject and a specific individual (Sara et al., 2012). Throughout the pairwise comparison method, subjects are able to make correct judgment and obtain more accurate results. However, since this scale requires seven figures, it hinders the application of the scale. Due to the limitation of research focus on interpersonal relationship, it is not applicable in organization management field. The IOS questionnaire allows subjects to select from various degrees of overlap between different circles to represent the individual-with-other relationship and to measure the psychological distance (Aron et al., 1992). In general conditions, this scale is used to assess the intimacy in romantic relationship (Uleman et al., 2000). However, recent studies also start to assess the intimacy of various interpersonal relationship (Woosnam, 2010). IOS questionnaire is a relatively new method to measure emotional intimacy in social psychology science. However, it is a single-item pictorial measure. Overall, most prior developments regarding psychological distance scales involved social situations, thus limiting their application area and circumstances. Because of the lack of precision and operability measurement tools, these scales cannot be directly applied to describing the employee-organization relationship. Despite these drawbacks, such studies represent valuable resources and contributed to the development of our scale.

The development of the EOPD scale is a creative work because few research results are available in this area of study. Prior studies yielded insufficient tools to analyze the complex system of EOPD. Therefore, Giddings and Grant (2006) proposed that when studying complicated issues such as individual thoughts and inherent emotions, it is more useful to combine several methods than to use a single method. Consequently, we combined the use of quantitative and the qualitative methods to develop the EOPD scale. In contrast, this study used interviews to develop the initial scale through qualitative analysis method and used investigation questionnaire data to quantitatively analyze the EOPD scale structure.

### Qualitative Method

#### Participants and Design

To extract the items for the initial EOPD scale, we first presented and conceptualized the reasons for and specific performances of the EOPD. We obtained the original items using the following methods:

We conducted targeted interviews of employees that included one psychological consultant, two college professors, one civil servant, one senior manager from a large-scale and state-owned company and five employees of private companies. We used Audacity software to record, direct and develop the output from the interviews.

We conducted an open-ended questionnaire on a sample of 300 employees who lived in different regions. A total of 277 valid questionnaires were collected; the valid return rate was 92.3%.

We reviewed existing domestic and international literature and used the systemic analysis method to analyze former theories and results regarding psychological distance and interpersonal relationships.

The interviews did not include preset patterns or pre-assumptions but did include a specific outline. This outline was used to guide interviewees by reviewing and describing relevant questions, which are provided in **Table 1** below. In addition, the open-ended questionnaire included the following statement: “The content of the answers is not limited; please answer the questions in as great detail as you can.” Moreover, the researcher was asked to communicate fully with the interviewees and to explain the interview prior to its onset.

**Table 1:** Outline of the EOPD interview.

Theme	Content
Basic information	Gender, age, marital status, monthly income, native place, work place, educational background, occupational area, type of work, nature of organization, positional hierarchy, positional grade, professional qualification.
Reason for the generation of EOPD	In daily life, age gaps, educational background, social class, religious faith, time spent-with, characters and values affect relationships between individuals. If we compare this to the employee-organization relationship, what do you think are the factors that affect the relationship between you and the organization that you work for?
Performance of EOPD	In daily life, numerous forms of the sense of distance are reflected in various aspects; when an individual feels close to someone, they will be considerate of him/her and wish to maintain a long relationship with that individual. However, when an individual feels distant from someone, they are unlikely to help that individual and may even reject him/her. If we compare this example to the employee-organization relationship, how would you describe the distance that you perceive between yourself and the organization you work for?

#### Ethical Approval

This study was carried out in accordance with the recommendations of *Ethical Codes of Consulting and Clinical Psychology of Chinese Psychological Society, Chinese Psychological Society*. The protocol was approved by the *China Occupational Safety and Health Association – Occupational Mental Health Professional Committee.* All subjects gave written informed consent in accordance with the Declaration of Helsinki.

It is the duty of researchers who are involved in psychological research to protect the life, health, dignity, integrity, right to self-determination, privacy, and confidentiality of personal information of research subjects. The responsibility for the protection of research subjects must always rest with our research team and *China Occupational Safety and Health Association – Occupational Mental Health Professional Committee* and never with the research subjects, even though they have given consent.

#### Procedure

**FIGURE 2:**
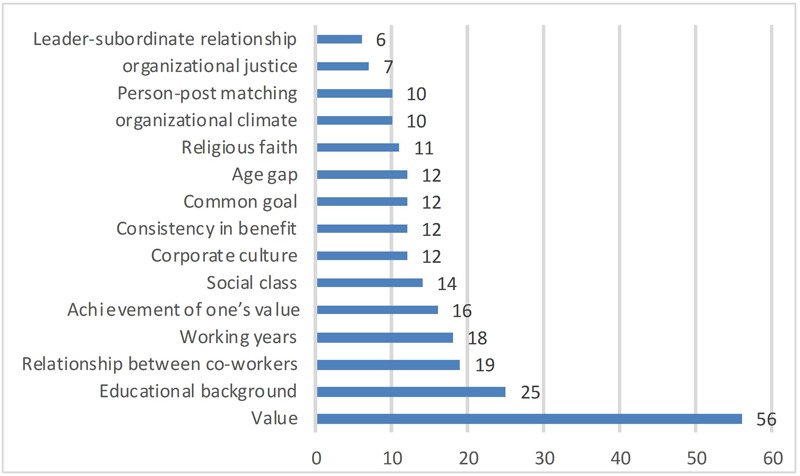
List of the classified items.

We use the CAQDAS software to categorize and segment qualitative data, which contains a large amount of text content to facilitate archiving and lookups, marking text fragments with Indep as a code, we invited five scholars in the field of business management to arrange the data that were collected from the interviews and questionnaires. Of these, 1133 expressions describing the EOPD were included. The researchers used labels to number the items. Later, by discussing these items, 103 ambiguous items (e.g., “objective feelings,” “realistic meanings” and “do what you ought to do”) were deleted. Finally, the researchers classified the remaining 1030 items and divided them into groups according to similarity and listed the items that frequently occurred as noted below in **Figure 2** counted through the CAQDAS program.

A total of 733 items remained after this reorganization of the data. Because the number of items was large, five researchers discussed the issue several times and decided to classify the items a second time according to their similarity in a semanteme. After the second step, 387 items remained. These items are provided in **Table 2** below.

**Table 2:** Classification of the semantically similar items.

Original statements	Conceptualization	Frequency
–I will protect organizational interests at the cost of my own interests when necessary; –I will give the biggest concession only if my own interest is not at stake; –I will decidedly resign and protect my legal rights using contract law.	Organizational benefit protection	40
–I think the level of affinity depends on the material security I obtain from the company (e.g., whether the salary fulfills my expectations); –I think the level of affinity depends on the amount of salary; –In regards to this question, I am neither close nor distant and have no particular willingness to stay or to leave; – I regard the relationship between myself and the organization as a monetary relationship; each of us takes advantage of the other for our own purposes.	Salary level	34
–When I feel close to the organization, it is reasonable for me to do anything for my group, and I am active in all types of work without becoming weary; –I only do the work that I was asked to do and do not actively care about other matters in the group.	Being active in work	31
–In regards to the goals of the organization, I will try my best and utilize all my resources to help my group overcome difficulties; –I only think about helping my organization when it does not go against my principles.	Organization assist	30
–I feel proud when my organization gets achievements; –I do not care about organizational matters, and it does not matter to me whether the organization progresses or not.	Share weal and woe	24
–I am happy with my organization, and I am content every day; –When I become distant from my organization, I want to quit and find a more suitable and enjoyable job regardless of how good the work conditions are; –I am happy about my career in education. My efforts at work are worthwhile as long as I can see the children’s smiling faces.	Happiness	16
…		

Because of the complexity of the above 387 items, we invited three additional professors and doctors to simplify the list by combining them based on a review of prior literature.

Similarity affects interpersonal psychology distance, the size of which is subject to a perceived (but not actual) similarity or dissimilarity between oneself and others. Berscheid and Walster (1978) noted that interpersonal attraction due to interpersonal similarity is rare between workers from different backgrounds; therefore, it is unlikely that cooperation will improve. Previous studies have indicated that numerous factors (including race, religion, manager-subordinate relationships, education, wealth, power, fame, economic status, age, and intelligence) affect social distance (Zhang, 2004). Based on the item collection and primary research, we classified the entries describing the characteristics (gender, age, educational background, social class, wealth, positional hierarchy, race, religion, IQ and EQ) of the employees into 10 scale items.

Increasing attention has been paid to employees’ emotions (Gooty et al., 2010); consequently, more studies have demonstrated that the establishment of a psychological relationship allows individuals to retain their emotional and physiological status at equal levels (Cwir et al., 2011). Specifically, individuals will experience the same mood as others, such as pleasure (Murray et al., 2002) and pain (Jackson et al., 2006). Kafetsios et al. (2014) noted that supervisors’ and subordinates’ insecure attachment orientations (higher anxiety and avoidance) were associated with own positive affect and satisfaction at work. For example, certain expressions were used to describe emotions including the following: “I adore my organization,” “I feel delight and enjoyment in my organization,” and “I share weal and woe with my organization.” Huang and Lin (2010) classified emotions into four dimensions: love, experience, sense of honor and sense of integration. These statements refer to the mentioned emotions in the following way: “I adore my organization” and “I feel delight and enjoyment in my organization” refer to “love”; “the organization is attractive to me” (along with the six items of “organizational identification”) and “the organization feels like home to me” (along with five items) refer to “experience”; “I feel sad when the organization is frustrated” and “I feel happy when the organization progresses” refer to “sense of honor”; “I care about the future of the organization,” “I like to share with my organization,” “I share weal and woe with my organization” and “I treat the organization’s affairs as my own affairs” refer to a “sense of integration.” Ultimately, 11 scale items were developed.

In addition, we noted that certain items were related to organizational citizenship behavior. Examples of these include the following: “willingness to take the initiative to join group discussions to provide suggestions for management and accomplish the work effectively and actively” [individual initiative (Podsakoff et al., 2000)], “willingness to assist the organization unconditionally” [assistant behavior (Podsakoff et al., 2000)], “obey the rules and regulations of the organization” [organizational compliance (Podsakoff et al., 2000)], “willingness to publicize and protect the organizational image” [loyalty to the organization (Podsakoff et al., 2000)], “willingness to improve oneself to contribute to the organization” [self-development (Podsakoff et al., 2000)], “willingness to have a correct view of, not to complain about organization” [sportsmanship (Podsakoff et al., 2000)] and “willingness to protect organizational benefit at the cost of self-benefit” [civil morality (Podsakoff et al., 2000)]. These behaviors may enhance organizational interests, and employees are willing to carry out these behaviors even if they are not given direct responsibility. In addition, these are behavior intention of embodying the affinity between employees and organizations (Bowler et al., 2010), representing a type of psychological closeness. Therefore, we separated these entries into 11 scale items.

Furthermore, we discovered that individuals are more likely to approach others who are considered to be members of their inner team. Conversely, individual spatial distances increase significantly for outer-team members (Liberman and Trope, 2014). Ying X. (2011) indicated that the sense of belonging to an organization and region predominantly influences an individual’s achievement motivation. Therefore, we combined the results of item frequencies and literature reviews to create seven scale items, including “my home is near the company,” “I know my position in the organization” and “I am willing to stay at the company even if I am off duty.”

We noted that the item “the degree of my comprehension of the organization” appeared only twice. However, previous studies have determined that familiarity has remarkable effects on psychological distance. For instance, familiarity may reduce the psychological distance between individuals and objectives and decrease the sense of danger and weaken the self-defense system, allowing individuals to feel more realistic, open and trustworthy (Pelham et al., 2005). Consequently, we introduced the item “I am quite familiar with my organization” into the scale to represent the level of an employee’s familiarity with their organization.

Based on a review of prior studies and numerous discussions regarding the 387 obtained items, the three experts reorganized, classified and extracted the expressions and obtained an EOPD scale that included 60 items. The purpose of our research is to enhance the theoretical logic and content validity of the EOPD system using a qualitative research method. The next step was to arrange and examine the EOPD system through quantitative data analysis.

### Quantitative Method

#### Preliminary Survey and Extraction of the EOPD Scale

The goals of the pre-survey were to estimate the quality of the initial questionnaires and to extract and modify the original items to obtain a formal EOPD scale.

##### Participants

In early June, 2016, we conducted an initial survey of employees who live in different regions. We used two methods, and the results were positive: 315 valid questionnaires were returned. The valid return rate was 82.9%. Later, we systemically analyzed the primary data and discovered the following findings: the gender ratio was balanced, 54% males and 46% females; the number of individuals whose education level included or exceeded a bachelor’s degree was 215 and accounted for 68.25% of the total sample; the age ratio was balanced, with 30.4% of the individuals below the age of 25, 21.36% between 26 and 30, 14.24% between 31 and 35, 13.27% between 36 and 40, 8.74% between 41 and 45, 7.12% between 46 and 50, and 4.87% older than 50. Furthermore, individuals in the sample were employed in a variety of industries (education, culture, finance, transportation, etc.) which indicated an acceptable level of representativeness for the sample. Ethical approval is same as “Qualitative method”.

##### Procedure

First, we examined the credibility of the initial questionnaire: We used Cronbach’s α factor to estimate the overall reliability of the scale. The output value of Cronbach’s α was 0.854 > 0.7, which indicated that the overall credibility was acceptable.

We used the project analysis method to estimate the credibility of every item. This analysis included four primary methods: (1) Missing values test. Three hundred fifteen candidates produced 188 omissions among 18,900 responses to 60 questions. The rate of missing values was 1%, of which 5% occurred in response to question 10 and 3.5% in response to item 2. (2) Descriptive statistical test. The descriptive statistical data indicate the basic quality of each project. The results indicated the following: the average values of items 2, 10, 12, 17, and 18 were distinctive (certain values were above 4.01, while others were lower than 3.01), and no item had a standard deviation of less than 0.75; an obvious skewness (coefficient of skew > 0.7) was indicated for items 2, 5, 10, 12, 17, 18, 19, 27, 28, 32, 39, and 46. (3) Comparison of extreme groups. We selected 27% of the employees from the highest score group and 27% from the lowest score group. This selection included 83 subjects whose score was either higher than 3.9883 or lower than 3.0117. These subjects were grouped together (as the extreme group) for testing. However, the results indicated that the T test value for item 2 did not reach 0.05, indicating that this item was not effective in identifying high or low values. (4) Homogeneity test. The results of a homogeneity test indicated that the coefficient of internal consistency was 0.976, and the homogeneity of the entire scale was high. However, the correlation coefficient and factor load value of items 2 and 6 were less than 0.3, and the factor loading value of item 17 was lower than 0.3. These items were not homogenous in our scale; therefore, we considered removing them from the scale. Moreover, all five indices of item 2 were are unsatisfactory and included “I do not think about this organization all the time, and I intend to quit in the future”; other items with three unsatisfactory indexes included items 10, 12, and 17. Therefore, we removed 12 items from the scale after the project analysis. Consequently, the value of Cronbach’s α increased to 0.902 from 0.854, and the number of items was reduced from 60 to 48.

Next, we conducted a principal components analysis on the remaining 48 items. We removed items that had a commonality of less than 0.5, a factor load value of less than 0.5 or a cross load value of greater than 0.4 through a times of factor analysis. We then removed items 7, 9, 11, and 26 and obtained a factorial structure with an acceptable level of discrimination. Consequently, we obtained an EOPD scale containing 44 items.

Then, we completed the item expressions through discussions with experts and by obtaining feedback from the interviewees to enhance the accuracy and clarity of the expressions and improve the content validity of the scale.

Finally, using a pre-survey, we ensured that the initial scale was of good quality and developed a formal EOPD scale that included 44 items; the questionnaire was edited for use in the final survey.

#### Formal Survey and Structural Analysis of the EOPD Scale

##### Data collection

In June, 2016, we collected data using questionnaires. Altogether 700 questionnaires were sent out and similar to the preliminary research, the research subjects were determined through the method of stratified sampling before the questionnaires issuing, covering different regions, different genders, different education level, different marital status and different occupation level, which can ensure the diversity, scientific quality and representative of samples. The questionnaires were issued to the designated research groups with a combination of internet-based and paper questionnaire, and we contacted the respondents and offered a detailed explanation of the research topic, research purposes and the related notes before the formal questionnaires issuing, which can ensure the return and effective rates. The total number of valid return was 554, and the valid usable return rate was 79.14%. In which gender, age, education, profession are well proportioned, and the occupation rates of marital status, organization character and post level are the reflection of the distribution in social reality. The specific sample distribution is represented on the **Table 3**. Ethical approval is same as “Qualitative method.”

**Table 3:** Sample distribution.

Sex	*N*	Marital status	*N*	Monthly income (RMB)	*N*	Positional grade	*N*
Male	278	Spinsterhood	189	<2000	137	No grade	347
Female	276	Married	355	2000–4000	101	Chief staff member	90
						
**Age**	***N***	Else	10	4000–6000	62	Section chief rank	44
					
<21	9	**Industry**	***N***	6000–8000	45	Department head rank	10
						
21–25	99	Agriculture, Forestry, Fishery and Husbandry	21	8000–10000	95	Minister Rank	8
26–30	75	Public Management	35	10000–30000	53	Else	55
						
31–35	79	Mining	112	30000–100000	39	**Positional Hierarchy**	***N***
						
36–40	89	Manufacturing	32	>100000	22	Ordinary	277
						
41–45	84	Construction	23	**Nature of organization**	***N***	First-line Manager	109
						
46–50	56	Retailing	15	Government	32	Junior Manager	81
51–55	41	Transportation	47	Public Institution	107	Senior Manager	54
>55	22	Catering	36	State-owned company	153	Else	33
					
**Diploma Level**	***N***	Information servicing	37	Collective Ownership Institution	18	**Professional Qualification**	***N***
					
Junior middle school and following	52	Finance	13	Private Company	102	No grade	241
Senior High School	72	Real estate	29	Sino-foreign Joint Company	46	Primary	95
Junior College	122	Education	80	Foreign-funded company	24	Junior	131
Bachelor Degree	187	Sanitary and Health	17	Joint-stock Company	50	Sub-senior	45
Master‘s Degree	92	Entertainment	44	Else	22	Senior	42
Ph.D. and Postdoctoral degree	29	Else	13				

##### Exploratory factor analysis

We used half of the sample data (277) to conduct an exploratory factor analysis of the scale using the statistical toolkit *SPSS 21.0.* The outcome value of KMO was 0.977 (>0.7), and the significance level was 0.000, which conforms to Bartlett’s test (*p* < 0.001). These results demonstrated that the formal scale was suitable for factor analysis. We then used Principal Components Analysis (PCA) and the Varimax Rotation method in the Orthogonal Rotation method to calculate the factor loads. Based on the Kaiser criterion, we selected six factors whose eigenvalues were higher than 1, and the accumulated variance contribution rate was 65.824%. All results are provided in **Table 4** below.

**Table 4:** Results of PCA and VR.

Item	Communality	Factor		Item	Communality	Factor			
		S1	S2			S3	S4	S5	S6
EOPD.42	0.785	0.754		EOPD.21	0.724	0.677			
EOPD.43	0.749	0.747		EOPD.27	0.725	0.646			
EOPD.47	0.741	0.746		EOPD.28	0.770	0.631			
EOPD.48	0.748	0.745		EOPD.22	0.761	0.618			
EOPD.40	0.732	0.744		EOPD.20	0.786	0.600			
EOPD.41	0.748	0.742		EOPD.23	0.702	0.575			
EOPD.44	0.732	0.736		EOPD.26	0.793	0.544			
EOPD.46	0.761	0.733		EOPD.25	0.747	0.542			
EOPD.45	0.733	0.725		EOPD.24	0.689	0.517			
EOPD.39	0.680	0.700		EOPD.14	0.582		0.671		
EOPD.32	0.689		0.709	EOPD.15	0.689		0.649		
EOPD.30	0.709		0.698	EOPD.17	0.703		0.615		
EOPD.33	0.731		0.673	EOPD.16	0.693		0.598		
EOPD.31	0.744		0.653	EOPD.13	0.487		0.562		
EOPD.34	0.731		0.614	EOPD.18	0.674		0.528		
EOPD.37	0.767		0.601	EOPD.2	0.382			0.763	
EOPD.35	0.721		0.588	EOPD.3	0.542			0.699	
EOPD.36	0.723		0.583	EOPD.1	0.556			0.650	
EOPD.38	0.729		0.554	EOPD.4	0.536			0.600	
EOPD.29	0.733		0.522	EOPD.6	0.608			0.521	
				EOPD.11	0.462				0.728
				EOPD.9	0.349				0.674
				EOPD.10	0.473				0.667

According to an analysis of the items of each dimension and previous studies, we defined six scale factors as provided in **Table 5** below.

**Table 5:** Definition of each factor.

Factor	Definition
Experiential distance	Employees’ perceptions regarding an organization’s future based on their assessment of an existing experience or trend
Behavioral distance	Employees’ perceptions regarding their affinity for an organization, which represents “beneficial to the organization” behavior
Emotional distance	Employees’ emotional perceptions regarding their correspondence or interactions with the organization.
Cognitive distance	Employees’ perceptions regarding their affinity for an organization regarding value orientation and personality consistency
Spatial-temporal distance	Employees’ perceptions regarding their affinity for an organization in space and time dimensions based on their level of involvement and understanding
Objective social distance	Employees’ perceptions regarding their affinity for an organization emerging from a similarity to population-based attributive characteristics

##### Confirmatory factor analysis

We used the remaining half of the sample data (277) to analyze the degree of fit between the observed data and a conceptual model using the confirmatory factor analysis method. To better test the accuracy of the model, we utilized six competing models and compared them with the previous results.

First, we developed six alternative models.

M1: Single factor model. We hypothesized that the 44 items had a common latent variable: EOPD.
M2: Double-factor model. We hypothesized that the 25 items of cognitive distance, emotional distance and behavioral distance have common latent variables and 19 items of spatial-temporal distance, experiential distance and objective social distance have common latent variables.
M3: Triple-factor model. We assumed that the 19 items of emotional distance and behavioral distance had common latent variables; 16 items of experiential distance and cognitive distance had common latent variables and 9 items of spatial-temporal distance and objective social distance had common latent variables.
M4: Four-factor model: We assumed that 19 items of emotional distance and behavioral distance have common latent variables; 16 items of experiential distance and cognitive distance have common latent variables; 5 items of spatial-temporal distance have common latent variables and 4 items of objective social distance have common latent variables.
M5: Five-factor model: We hypothesized that 19 items of emotional distance and behavioral distance have common latent variables; 6 items of cognitive distance have common latent variables; 10 items of experiential distance have common latent variables; 5 items of spatial-temporal distance have common latent variables and 4 items of objective social distance have common latent variables.
M6: Six-factor model: We assumed that emotional distance, behavioral distance, cognitive distance, experiential distance, spatial-temporal distance and objective social distance are factors in this model.

Next, we regarded each factor as a latent variable and regarded the items of each factor as observational variables in every model to test their model fit (**Table 6**) using the confirmatory factor analysis method. The results demonstrated that models M1, M2, M3, M4, and M5 resulted in poor fits. In addition, the GFI and AGFI values of these models were all less than 0.7, the values for NFI, CFI, TLI and IFI were all less than 0.9, and the RMSEA values were all greater than 0.07. In comparison, the χ^2^/df value of M6 was the lowest of the six tested models (2.698). Furthermore, the NFI, CFI and IFI values of M6 were all greater than 0.9. Therefore, we concluded that Model 6 yielded the best results, although certain indices were unsatisfactory. Later, we modified the model parameters; the variance coefficients for which the modified index was greater than 20 are listed in **Table 7**.

**Table 6:** Major fitting degree indices of employee-organization psychological distance.

Model	*χ*^2^	df	χ^2^/df	GFI	AGFI	NFI	CFI	TLI	IFI	RMSEA
M1: Single factor model	4733.9	902	5.248	0.569	0.527	0.717	0.758	0.746	0.758	0.097
M2: Double-factor model	3884.8	901	4.312	0.650	0.615	0.768	0.811	0.802	0.812	0.086
M3: Triple-factor model	3777.6	899	4.202	0.653	0.618	0.774	0.818	0.808	0.818	0.084
M4: Four-factor model	3565	897	3.974	0.659	0.624	0.789	0.826	0.814	0.827	0.079
M5: Five-factor model	3086	895	3.448	0.678	0.636	0.807	0.837	0.822	0.837	0.071
M6: Six-factor model	2409.4	893	2.698	0.814	0.891	0.903	0.904	0.898	0.904	0.059

**Table 7:** Overall fitting degree indices of each modification.

Title		Initial model fitting	Release e13–e18	Release e27–e28	Release e1–e2	Release e39–e40	Release e9–e10	Assessment
Absolute fitting index	χ^2^	2409.351, df = 893 *P* = 0.000	2387.274, df = 891 *P* = 0.000	2369.125, df = 889 *P* = 0.000	2301.865, df = 886 *P* = 0.000	2236.437, df = 883 *P* = 0.000	2123.155, df = 881 *P* = 0.000	Great
	GFI	0.814	0.824	0.848	0.854	0.891	0.901	Great
	RMR	0.312	0.301	0.296	0.263	0.243	0.224	Poor
	RMSEA	0.059	0.057	0.054	0.052	0.050	0.048	Good
Relative fitting index	AGFI	0.891	0.894	0.896	0.898	0.890	0.902	Great
	NFI	0.903	0.905	0.907	0.909	0.911	0.913	Great
	TLI	0.898	0.901	0.905	0.909	0.912	0.916	Great
	CFI	0.904	0.908	0.912	0.916	0.920	0.922	Great

*GFI, Goodness of Fit Index; RMR, Root Mean Square Residual; RMSEA, Rood Mean Square Error of Approximation; AGFI, Adjusted Goodness of Fit Index; CFI, Comparative Fit Index; TLI, Tucker-Lewis Index; NFI, Normed Fit Index.*

**FIGURE 3:**
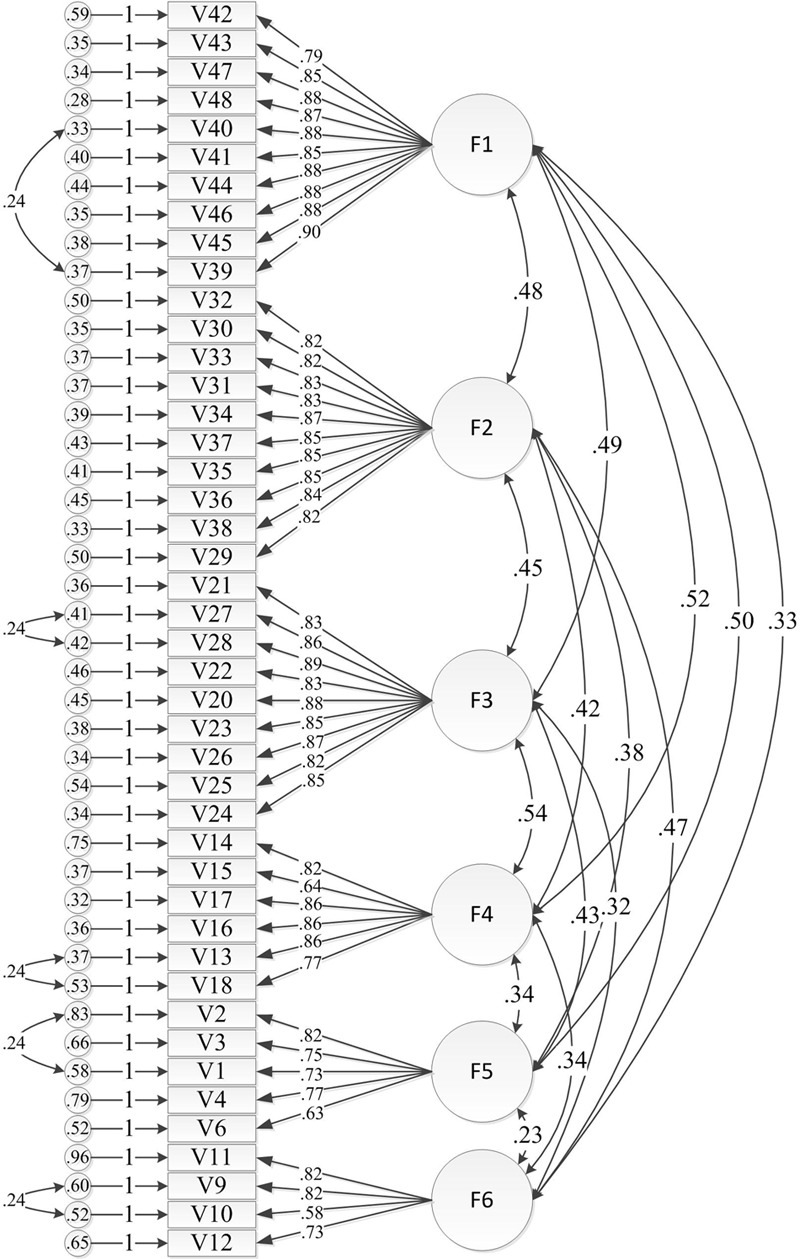
Estimations of the standardized path coefficient of the final confirmatory factor model.

Finally, the GFI, AGIF, NFI, TLI and CFI values were greater than 0.9 after five modifications, the RMSEA value was less than 0.05, and the χ^2^/df value was decreased to 2.410. The finding of an acceptable level for each index indicated that the EOPD model yielded an ideal fit. The standardized factor correlation coefficients remained at 0.30, indicating that the items were reasonably designed. The standardization method is provided in **Figure 3**.

##### Reliability and validity

A reliability assessment was conducted on the entire scale and all latent variables. We used Cronbach’s alpha value (>0.7) to test the overall reliability of the scale and used both Cronbach’s alpha and the CR value (>0.6) to test the reliability of latent variables. The overall reliability of the EOPD scale was found to be 0.971 (Cronbach’s alpha value), indicating that the scale was acceptably reliable. The reliability values of each latent variable were in the range from 0.737 to 0.956, and the CR values were in the range from 0.829 to 0.968. These results illustrated that both the overall reliability of the EOPD scale and that for each latent variable were acceptable.

The scale was evaluated for content validity and structure validity. Content validity may be controlled using qualitative methods; however, structure validity primarily tests convergent validity and discriminant validity. This research strictly followed standard scale development procedures and implemented the following steps: First, based on previous studies, we collected original items using interviews and 277 open-ended questionnaires. The sample exhibited good universality and pertinence during this step. Second, we invited five researchers and three experts in the business management field to discuss question design. Third, we conducted 315 pre-surveys to ensure content validity. The standardized factor loads of the latent variables of 44 scale items were higher than 0.5 and reached significance; AVE values were in the range from 0.553 to 0.751 (>0.5) and reflected a satisfactory degree of convergence. The square roots of the AVE values of these latent variables were higher than the correlation coefficients between them, indicating an acceptable degree of structural discrimination. Therefore, the scale fulfilled the validity test criteria. The results are provided in **Table 8**.

**Table 8:** Estimations of the standardized path coefficient of the final confirmatory factor model.

Factor model	F1	F2	F3	F4	F5	F6
F1	0.867^∗^					
F2	0.611	0.839^∗^				
F3	0.580	0.409	0.854^∗^			
F4	0.322	0.444	0.453	0.803^∗^		
F5	0.402	0.423	0.302	0.356	0.743^∗^	
F6	0.336	0.491	0.522	0.387	0.401	0.744^∗^
Cronbach’s α	0.956	0.953	0.940	0.876	0.833	0.737
CR	0.968	0.960	0.960	0.915	0.860	0.829
AVE	0.751	0.704	0.729	0.645	0.552	0.553

*^∗^Represents the square root of AVE value.*

## Conclusion and Limitations

### Discussion

The measurement of employee-organization relationship with EOPD scale from the dimension of objective social distance, cognitive distance, emotional distance, behavioral distance, experience distance and time distance has objective rationality and it authentically and explicitly shows the distance of employee-organization relationship. Bar-Anan et al. (2006), Trope and Liberman (2010) on psychological distance, from the perspective of construal level research can be divided into space distance, time, distance, social distance and hypotheticality, though not in the organization of interpersonal problems, but in the description of the structure of psychological distance and this study has higher consistency, thus contributing to comprehensively knowing about employee-organization relationship and elevating organization management efficiency.

Relational similarity is a form of social distance, Liviatan et al. (2008) similarity to others by examined the impact of interpersonal similarity to representation and judgment of others’ actions and found that compared with similar targets, the participants with similar targets would improve its behavior characterization to the construction of higher level, interpersonal similarity information processing is of great significance for others. Giacomantonio (2010) studied how psychological distance and social interaction influence the behavior of individuals in the social decision-making environment, and argued that “increasing pro-social” “increased pro-sociality” is conducive to solving social conflicts. This study considers that employees will generate intimacy perception difference toward the organization based on similarity difference of group characteristics. In another word, employee-organization objective social distance is able to represent employee-organization relationship.

Individual-organization matching indicates the consistency and compatibility between employee and organization in values (Kristof, 1996), while value matching signals the consistency between employee and organization in culture, objective, atmosphere and other characteristics (Cable and Judge, 1994). As an important content in employee-organization matching (Verquer et al., 2003), value matching allows employees to establish a more intimate relationship with organization and mirror organization characteristics and values to self-concept and self-definition. In this way, employees would define themselves as members within the organization (Vilela et al., 2008) and draw close the psychological distance with organization. Employee-organization matching degree influences the intimacy perception of employee toward organization. Wu and Bai (2015) pointed out that in the study of person-organization fit which directly affect the psychological distance, but not directly to the organization “integrated force (integrated force),” but through the psychological distance. As a result, the cognitive distance between employee and organization is able to reveal employee-organization relationship.

The affection of employee toward organization directly reflects employee-organization relationship (Cwir et al., 2011). Positive or negative psychological experience and emotional experiences of employees within the organization are important factors used to predict employee behaviors and performance (Özaralli, 2003). In another word, employees’ emotional intimacy and perception with organization would influence employee-organization relationship.

Organization citizen behavior has turned to be one of the most important work inputs of employees within the organization (Podsakoff et al., 2000) and becomes supportive organization management practice behaviors (Snape and Redman, 2010). The stable and emotional connection between employees and organization (leaders) has potential function to propel emotional exchange, enhance employee’s internal cognitive identity (Wang et al., 2009) and elevate employee’s satisfaction (Lapierre and Hackett, 2007). This implies that employees would like to perform more behaviors outside job duties for the improvement of organization benefits (Bowler et al., 2010). Instead, if an employee would like to positively work for the organization and perform pro-organization behaviors outside job duties, this employee has sense of belonging and identity in the organization. In another word, employee-organization behavioral distance could directly reflect the intimacy between employee and organization.

Employees would acquire the perception about future expectations of the organization based on present conditions or tendency judgment experience, while such perception directly influences employee-organization relationship. This conclusion has relatively high consistency with existing studies. Employees usually pay more attention to future development prospects in the organization, including promotion chance (Kraimer et al., 2011), salary treatment (Carraher, 2011; Li et al., 2016) and all other things referred to as expectations. This determines employee’s satisfaction and work state in the organization to a large extent. In another word, the expected distance between employee and organization could reflect the relationship between the two.

The working year and space distance of employees within the organization directly determine their understanding and participation degree toward the organization. As proved by some studies, employees usually take a series of factors into consideration during employment and resignation process, including region (Ying X.H., 2011), time length to get along with organization (Zhang and Liu, 2009) and so on. Such regional affiliation and emotional fetters would produce influences on the relationship between employees and organization. Lim et al. (2012) explored psychological distance between social media services users in the research of Social media, and found that living space inhabited space can reduce the psychological distance between the users, Gulyas (2016) pointed out that the space distance between team members is associated with greater psychological distance, spatial distance and psychological distance between the team members cooperation and trust, which has higher consistency with this study. This proves that employee-organization time space could reflect the relationship between the two.

### Conclusion

This research used a qualitative analysis method to develop an initial EOPD scale. The data were collected from a review of previous studies, in-depth interviews with 10 employees and 277 open-ended questionnaires. Project analysis and principal component analysis were then used to examine and extract the items to develop an EOPD scale containing 44 items. The data were collected from 315 pre-surveys.

We collected 554 formal investigation questionnaires and used half of them in the principal components analysis and finally gained six factors (experiential distance, behavioral distance, emotional distance, cognitive distance, spatial-temporal distance, and objective social distance). The analyzing result contained: KMO value of 0.977 (>0.7), the significance degree of 0.000 and the accumulated variance contribution rate of 65.824%. Then we used confirmatory factor analysis to analyze data from the rest 277 questionnaires and found out the optimal model M6 from six models. The modified results contained: the value of GFI, AGIF, NFI, TLI and CFI corresponding to 0.901, 0.902, 0.913, 0.916 and 0.922 respectively, the RMSEA value of 0.48 and the χ2/df value of 2.410. All the indices had reached a decent level which reflects that the EOPD scale has a satisfactory fitting degree.

The Cronbach’s alpha which reflects the overall reliability of the scale is 0.971, along with the value of each latent variables of 0.956, 0.953, 0.940, 0.876, 0.833, 0.737, the CR value of 0.968, 0.960, 0.960, 0.915, 0.860, 0.829, respectively. These results meant that the scale met the validity test criterions. Moreover, the scale was developed according to a strict procedure, which can ensure its scientificity and precision. The square roots of AVE values of these latent variables were higher than the correlation coefficients, while the relative AVE values of their construct validity were 0.751, 0.704, 0.729, 0.645, 0.552, and 0.553 (>0.5). Therefore we can determined that the EOPD scale possesses a decent level of reliability.

### Limitations and Future Studies

This study had certain limitations. First, the geographical regions within which the individuals were sampled were limited. Although we attempted to obtain typical and representative information and included both developed and undeveloped geographical areas, certain provinces or municipalities were not included in this study (e.g., Taiwan). Furthermore, respondents’ answers to questions relied on memories that may have been affected by memory biases. Additionally, it was difficult to construct theories because psychological distance is an objective perception. Therefore, improvements and modifications are needed in future research.

The objective of this study was to develop an EOPD scale for use in management practice. We expect that more studies can be designed to analyze specific individual characteristics, work characteristics and organizational characteristics using the EOPD scale. Furthermore, psychological distance may be introduced as a moderator in research areas such as occupational burnout, turnover intention, work-embedded or adverse selection and may improve the effects of business management. In future research work, additional cross-sectional studies are needed. Throughout the comparison with other psychological intimacy scales, the researcher could empirically test that their scale has more predictive power than other previous measures of psychological closeness as for instance the IOS scale and further verify the superiority of EOPD scale. In future research work, additional longitudinal studies are needed. Therefore, next step of the study is to organize multiple investigations with research subjects at different time intervals so as to verify the time stability of the scale.

## Author Contributions

HC designed the frame of this paper and wrote the paper; SL performed the field research and analyzed the data.

## Conflict of Interest Statement

The authors declare that the research was conducted in the absence of any commercial or financial relationships that could be construed as a potential conflict of interest.
